# Pheno‐Morphological Screening and Acoustic Sorting of 3D Multicellular Aggregates Using Drop Millifluidics

**DOI:** 10.1002/advs.202410677

**Published:** 2025-01-10

**Authors:** Leon Rembotte, Thomas Beneyton, Lionel Buisson, Amaury Badon, Adeline Boyreau, Camille Douillet, Loic Hermant, Anirban Jana, Pierre Nassoy, Jean‐Christophe Baret

**Affiliations:** ^1^ CNRS Univ. Bordeaux CRPP UMR 5031 Pessac F‐33600 France; ^2^ LP2N Univ. Bordeaux Talence F‐33400 France; ^3^ IOGS CNRS UMR5298 Talence F‐33400 France; ^4^ TreeFrog Therapeutics Pessac F‐33600 France

**Keywords:** acoustics, microfluidics, organoids, screening, sorting

## Abstract

Three‐dimensional multicellular aggregates (MCAs) like organoids and spheroids have become essential tools to study the biological mechanisms involved in the progression of diseases. In cancer research, they are now widely used as in vitro models for drug testing. However, their analysis still relies on tedious manual procedures, which hinders their routine use in large‐scale biological assays. Here, a novel drop millifluidic approach is introduced to screen and sort large populations containing over one thousand MCAs: ImOCAS (Image‐based Organoid Cytometry and Acoustic Sorting). This system utilizes real‐time image processing to detect pheno‐morphological traits in MCAs. They are then encapsulated in millimetric drops, actuated on‐demand using the acoustic radiation force. The performance of ImOCAS is demonstrated by sorting spheroids with uniform sizes from a heterogeneous population, and by isolating organoids from spheroids with different phenotypes. This approach lays the groundwork for high‐throughput screening and high‐content analysis of MCAs with controlled morphological and phenotypical properties, which promises accelerated progress in biomedical research.

## Graphical abstract

ImOCAS: a millifluidic system designed for large‐scale analysis and sorting of 3D multicellular aggregates (MCAs) like spheroids and organoids. It rapidly screens hundreds of samples based on their phenotypes and morphologies by combining real‐time image processing with acoustic actuation of liquid drops, enabling high‐throughput analysis of 3D biological samples with potential applications in regenerative medicine.

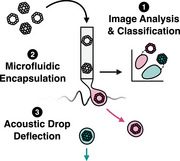

## 1 Introduction

Over the past decade, three‐dimensional (3D) multicellular aggregates (MCAs) have emerged as the new gold standard to investigate fundamental cell biology processes with a higher degree of physiological relevance compared with two‐dimensional (2D) cultures.^[^ ^1^ ^]^ In particular, key phenomena like gene expression,^[^ ^2^ ^]^ cell–cell, and cell–matrix interactions,^[^ ^3^ ^]^ physiology^[^ ^4^ ^]^ and differentiation^[^ ^5^ ^]^ have been shown to be better recapitulated in 3D models. The versatility of 3D models extends to a wide range of applications. In cancer research, multicellular spheroids, potentially composed of several cell types, serve as in vitro tumors for disease modeling and drug testing.^[^ ^6^ ^]^ In cell therapy and regenerative medicine, organoids derived from patient cells or induced pluripotent stem cells (iPSCs) are used as building blocks to repair tissues.^[^ ^7^ ^]^ Recently, organoids have even been validated as an alternative to animal testing as stated in the US Food and Drug Administration Modernization Act 2.0.^[^ ^8^ ^]^ This ever‐growing interest for 3D cell models in biological studies pushes for the development of standardized methods to produce, analyze, and screen them.

The prerequisite for the production of 3D cell models is cell self‐organization in a controlled environment.^[^ ^9^ ^]^ Traditional production methods such as the hanging drop^[^ ^10^ ^]^ and the spinning culture^[^ ^11^ ^]^ have proven efficient for the formation of MCAs, yet they demand considerable time investment, which is incompatible with experiments involving hundreds of samples. In contrast, microfluidic approaches enable high throughput production of MCAs.^[^ ^12^ ^]^ Among others, the Cellular Capsules Technology (CCT) was designed to encapsulate cells in hollow alginate shells, allowing them to self‐assemble into MCAs and grow in confined, niche‐like microenvironments, hence producing thousands of MCAs per second.^[^ ^13^ ^]^ More broadly, recent developments in the field of microfabrication led to increased control over the production of MCAs and offered the possibility to tune the chemical and mechanical properties of their microenvironments.^[^ ^14^ ^]^ All these methods however inherently introduce variability in the initial cell seeding densities and nutrient concentrations, which subsequently gives rise to considerable sample heterogeneity in size and phenotype.^[^ ^15^ ^]^ In this context, while most efforts were initially devoted to the production of MCAs, the bottleneck in the field of 3D biology has now shifted toward automated, high‐throughput characterization, and manipulation methods.

The analysis of MCAs most often relies on user‐dependent, manual methods. The key challenges result from their 3D nature and their wide range of sizes, from 50 µm to 5 mm in diameter.^[^ ^16^ ^]^ A widespread approach consists in the dissociation of MCAs to perform single‐cell analysis, or even in the lysis of the cells to perform biochemical assays.^[^ ^17^, ^18^ ^]^ These methods are however highly destructive and come with a complete loss of structural information. To analyze MCAs while preserving their integrity, optical microscopy remains the most adapted tool, since it allows to study MCAs at the multicellular level with a sub‐cellular resolution.^[^ ^19^ ^]^ For instance, the growth dynamics and 3D internal organization of MCAs were unraveled thanks to advances in depth‐resolved fluorescence microscopy,^[^ ^20^ ^]^ and in multi‐plane image segmentation algorithms.^[^ ^21^ ^]^ Due to their complexity, these high‐content approaches may only be applied to a small number of MCAs at a time. On the contrary, microfluidics made it possible to immobilize hundreds of MCAs in microwells and perform time‐lapse imaging at the population level, although at the cost of decreased resolution.^[^ ^22^, ^23^ ^]^ Recently, developments in light‐sheet fluorescence microscopy additionally provided the possibility to perform high‐content analysis of numerous MCAs in microengineered wells,^[^ ^24^ ^]^ or in manually controlled flow.^[^ ^25^ ^]^ However, none of these approaches allows to manipulate MCAs for sorting purposes. Using MCAs in drug testing or disease modeling requires rapid screening of large populations of MCAs to gather statistically relevant information, and to isolate MCAs of interest for further analysis. To date, this would only be feasible through tedious pipetting of samples and time‐consuming image acquisition.

Inspired by the development of flow cytometry in the field of single‐cell analysis, we develop a flow‐based approach to address the pressing need for automated manipulation of MCAs. Since its invention in 1965,^[^ ^26^ ^]^ flow cytometry has been massively adopted in biology facilities, especially through the advent of fluorescence activated cell sorting (FACS) for single‐cell analysis.^[^ ^27^ ^]^ In flow cytometry, a suspension of cells continuously flows through a capillary where parameters of interest are measured in individual cells. They are then encapsulated into liquid drops by passing through a vibrating nozzle. Finally, the drops are deflected on‐demand by submitting them to an electric field while they fall. However, the adaptation of flow cytometry to the analysis of MCAs has hardly been explored, mainly because of the clogging risks that arise when working with such large, weakly deformable objects. To our knowledge, only one group reported in 1987 the modification of a commercial flow cytometer to sort spheroids by increasing the size of the exit nozzle, hence allowing to study MCAs smaller than 100 µm in diameter.^[^ ^28^ ^]^ Despite its pioneering nature, this approach was limited by the small size of the MCAs it could sort and its compatibility solely with detection techniques specific to standard FACS.

In addition to the challenges related to fluidics, scaling up from single cells to whole MCAs requires to redefine the parameters of interest that need to be measured. A classical analysis based on fluorescence intensity or light scattering cannot satisfactorily be used to characterize thick 3D objects. It would imply measuring averaged parameters over the whole MCAs, which comes with a loss of structural information. On the contrary, forming an optical image of a MCA gathers information across a whole surface, which yields a more complete description of their spatial organization. Such image‐based approaches have only recently been unlocked for single‐cell analysis using microfluidic devices.^[^ ^29^, ^30^, ^31^ ^]^ Not only microfluidic systems are suitable for imaging biological systems, but they are also compatible with many actuation methods previously implemented for single‐cell sorting: electrophoresis,^[^ ^32^ ^]^ dielectrophoresis,^[^ ^33^ ^]^ acoustophoresis,^[^ ^34^ ^]^ optical manipulation,^[^ ^35^ ^]^ or mechanical actuation.^[^ ^36^ ^]^ Recent developments in drop‐based microfluidics further increased the versatility and the throughput of cell sorting by miniaturizing the principle of FACS into micrometric channels.^[^ ^37^ ^]^ Again, these approaches cannot be adapted for MCAs simply by increasing the channel dimensions. The large size of MCAs leads to a more predominant role of inertial forces, together with increased risks of sedimentation and clogging compared to single cells. For these reasons, the automated sorting of MCAs in a flow‐based microfluidic device has hardly been explored yet. To the best of our knowledge, only one team recently demonstrated the separation of polystyrene beads from spheroids using image processing, at a rate of 0.2 Hz.^[^ ^38^ ^]^

To address the limitations that currently prevent the widespread use of MCAs, we introduce a novel drop‐based approach to perform Image‐based Organoid Cytometry and Acoustic Sorting (ImOCAS). Like classical flow cytometers and droplet microfluidic devices, it carries out three primary operations: detection of a feature of interest in MCAs in flow, encapsulation of individual MCAs in liquid drops, and actuation of the drops of interest. Here, these steps were redesigned to allow the manipulation of biological samples up to several hundred microns in diameter. In ImOCAS, spheroids and organoids are continuously flowed through a square glass capillary where their morphological and phenotypical signatures are characterized on‐the‐fly using bright‐field microscopy image analysis. They are then individually encapsulated in millimetric drops of culture medium which are sorted on‐demand using the acoustic radiation force (ARF) generated by a standing‐wave acoustic field.

To illustrate the capabilities of ImOCAS, we screen large populations exceeding one thousand MCAs to extract statistical distributions of morphological and phenotypical features. We demonstrate its ability to accurately select spheroids of the same size from heterogeneous populations, which is a necessary initial step for subsequent drug testing. We also show its capacity to classify and separate plain MCAs from those containing a hollow core (lumen), which is a complex and time‐consuming task when performed manually. The versatility, simplicity, and generality of ImOCAS suggest its widespread adoption in 3D biology laboratories, with the potential to accelerate drug discovery and fundamental research thanks to high‐throughput morphological and phenotypical screening of spheroids and organoids.

## 2 Results

### 2.1 Operating Principle of ImOCAS

**Figure 1:**
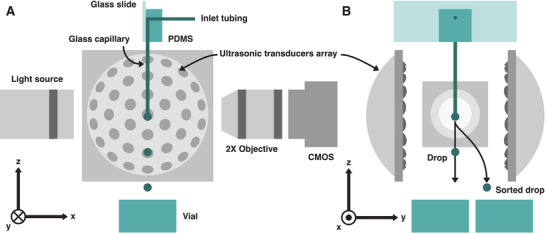
Schematics and working principle of ImOCAS. Side views of the system in the *xz*‐ plane (A) *yz*‐plane (B). Arrows indicate schematic drop trajectories. All elements are at scale (except the capillary which is twice wider), but the distance between the objective and the CMOS is shortened.

We first briefly explain the working principle of ImOCAS before detailing the challenges to overcome at each step in the following sections. **Figure** **1** shows a schematic view of our system, which comprises three modules for the detection (i), encapsulation (ii) and actuation (iii) of MCAs.

(i) Opto‐fluidic detection. Real‐time imaging and analysis of MCAs is required to characterize them with human‐interpretable features. Since ImOCAS relies on label‐free imaging, no particular sample preparation is required. MCAs are dispersed in a culture medium solution and flowed through a square glass capillary, which is preferred to a cylindrical glass capillary to avoid optical aberrations (Figure 1A). Micrographs are taken and analyzed on the fly at ≈100 frames per second (fps) to measure the morphological and phenotypical features of MCAs, which are then compared with user‐defined criteria to make a sorting decision. (ii) MCA encapsulation. Each MCA is encapsulated in a millimetric drop of culture medium at the capillary exit. As detailed in Section 2.1, the concentration of MCAs is optimized to encapsulate only one per drop, and to prevent the clogging of the exit capillary. (iii) Acoustic actuation. The goal is to deflect individual drops using the ARF without altering the flow. A standing‐wave acoustic field is generated by two arrays of ultrasonic transducers operating at 40 kHz. The arrays consist of two spherical caps, with their focal point placed close to the capillary exit (Figure 1B). Upon detection of a target MCA, a trigger signal is sent to a waveform generator to activate a standing‐wave acoustic field in proximity of the capillary exit. This deflects the very next drop toward a collection vial, while nondeflected drops are collected separately.

### 2.2 Image‐Based MCA Screening with On‐the‐Fly Detection of Pheno‐Morphological Features

Flow cytometry requires the measurement of a well identified set of parameters in a large number of objects, both for the collection of statistically relevant data and for the identification of potentially rare events. Here, we specifically aim at measuring morphological and phenotypical attributes of MCAs. More specifically, we analyze their sizes and shapes as first order discriminating parameters, and we monitor the presence or the absence of a lumen, which is a phenotypic property of epithelial tissues. We introduce a pipeline for rapid image‐based characterization of MCAs, comprising: the continuous acquisition of bright‐field, monochromatic images upstream from the glass capillary exit, the detection of an MCA via binary image processing, the quantification of its morphological and phenotypical features, and finally its classification as target or waste based on user‐defined criteria. Among the variety of shapes and topologies found across multicellular aggregates, we focus on two models: spheroids (plain ellipsoidal aggregates) of immortalized human embryonic kidney cells (HEK), very often encountered in tumor models, and cysts (spherical monolayers of epithelial cells surrounding a lumen) of induced pluripotent stem cells (iPSCs), from which organoids are often derived. Here, both spheroids and cysts are formed in hollow hydrogel shells using the CCT technique (see Experimental Section).

**Figure 2:**
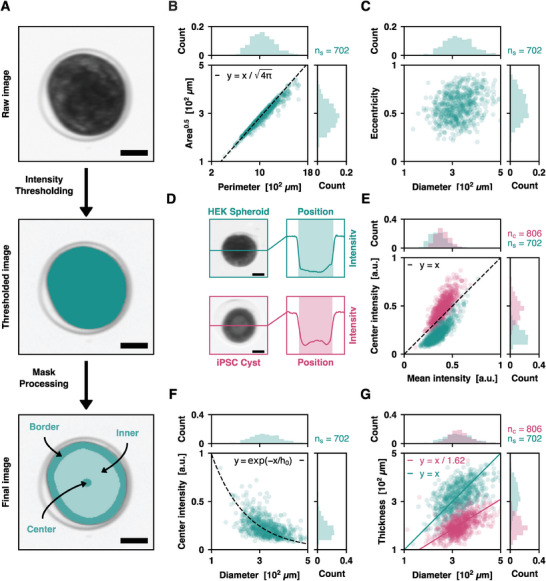
On‐the‐fly image processing for MCA classification. A) Principle of the image processing pipeline. The raw image is normalized with a reference background image, which enables to detect the MCAs using automated intensity thresholding. Binary mask processing (erosions and dilations) is used to define three regions of interest—the Center, the Inner, and the Border regions. The hydrogel shell containing the cells is visible but is not considered in the analysis. Scale bars: 100 µm. B) Plot of the square root of the area versus perimeter of HEK spheroids. The broad distribution of sizes results from variations in initial cell densities during the production of spheroids. *n*_s_ = 702 spheroids. C) Plot of the eccentricity versus diameter of HEK spheroids. This refined morphological characterization enables to detect target MCAs with a given sphericity. *n*_s_ = 702 spheroids. D) Transmitted intensity profiles in HEK spheroids and iPSC cysts. A bright center spot is observed in cysts due to the presence of a weakly absorbing lumen, whereas the increased cell density at the center of spheroids results in a minimum of intensity at the center. Scale bars: 100 µm. E) Plot of the intensity at the center versus mean intensity for iPSC cysts (pink) and HEK spheroids (blue). The two populations are separated by the *y* = *x* line. *n*_c_ = 806 cysts, *n*_s_ = 702 spheroids. F) Plot of the intensity at the center versus diameter for HEK spheroids. Fitting a linear model to Beer's law with a linear model yields a typical attenuation length *h*_0_ = 1.4 ± 0.1 × 10^2^ µm. *n*_s_ = 702 spheroids. G) Plot of the estimated tissue thickness versus diameter for HEK spheroids and iPSC cysts. Using the value of *h*_0_ obtained with spheroids, we estimate the tissue thickness by inverting Beer's law. For cysts, we find a linear scaling law with *h = D/1.62*. *n*_c_ = 806 cysts, *n*_s_ = 702 spheroids.

**Figure** **2**A summarizes the image processing steps performed in ImOCAS. A user‐based intensity threshold is applied to each bright‐field image to obtain a binary mask corresponding to the MCA. The projected area of the analyzed MCA is then simply derived by counting the number of pixels (px) in the mask. An additional step of mask morphology processing (erosions and dilations) enables to define three regions: Center, Inner, and Border. The perimeter of the MCA is calculated by counting the number of pixels in the Border region. The Inner region is used to compute the mean pixel intensity across the MCA without being affected by irregularities close to the edges. Additionally, we use built‐in Python libraries to fit an ellipsoid to the mask and measure the eccentricity of the MCA. The whole pipeline is compatible with on‐the‐fly analysis of ≈100 fps in 256 × 256 px^2^ images (Figure S1A, Supporting Information).

To validate this morphological analysis, we first measure the distribution of sizes in a population of HEK spheroids (Figure 2B). This operation is required in toxicity assays, where MCAs of well‐defined sizes must be used. We observe that the areas *A* and perimeters *P* of spheroids are correlated with a scaling law *A* ^0.5^ = *k* × *P*. At the population level, we find as a first approximation *k* = (4π)^0.5^, which is the expected value in the case of perfectly spherical objects. This legitimates the definition of an equivalent diameter *D =* 2 × (*A* / π)^0.^ ^5^, yielding a mean spheroid diameter *D* = 3.3 ± 0.6 × 10^2^ µm. The observed size polydispersity arises from stochastic variations in initial cell seeding concentrations, which are inherent to every MCA formation technique. This polydispersity tends to increase over time, as the number of cells grows exponentially. Apart from their sizes, spheroids may also differ in shape, as they are not necessarily spherical. They are appropriately characterized by their eccentricity *e*, defined by the relation *e* ^2^ = 1 – *b* ^2^ / *a* ^2^, where *b* and *a* are the semi‐minor and semi‐minor axis obtained with an ellipse fit (Figure 2C). We measure a mean eccentricity *e* = 0.57, corresponding to a 17% relative variation between *a* and *b*. Here, *e* is actually an apparent eccentricity, because the image is the projection of an ellipsoid on the imaging plane, which depends on the orientation of the spheroid with respect to the camera. In the present case of spheroids produced in hydrogel shells, this eccentricity mainly originates from the very shape of the shells to which the spheroids conform upon reaching confluence. From a more general perspective, *e* may be used as a marker to study the anisotropic growth of MCAs, as observed typically in gut organoids.^[^ ^39^ ^]^ Overall, this morphological approach allows to measure quantitative shape‐related parameters in large populations of spheroids, which is utilizable for subsequent sorting of spheroids with a target size or shape.

We then extend the methodology to a phenotype‐driven analysis. Here, spheroids are distinguished from cysts through the analysis of the radial intensity profile of light transmitted through the MCAs (Figure 2D). In the Border region, the intensity of a cyst and a spheroid are similar because they are equally composed of cells. In contrast, their intensities in the Center region differ due to unequal cellular contents. In the case of spheroids, the length of absorbing material through which photons pass is maximum at the center. This corresponds to a minimum of transmitted light due to increased absorption and scattering. Conversely, in cysts, the deficit of absorbing material in the lumen results in a local maximum of transmitted light in the vicinity of the center. Therefore, we discriminate cysts from spheroids by comparing the center intensity to the mean intensity, where the center intensity is taken as the mean intensity over a small disk of 11 px in diameter (empirically chosen) around the centroid, and the mean intensity is taken as the mean intensity within the Inner region. Figure 2E shows that cysts and spheroids are found, respectively, above and below the threshold line where center and mean intensities are equal, which validates the relevance of this discrimination criterion based on an explainable, physical argument. Altogether, this approach yields 99% precision (true positive/true positive + false positive) and 90% recall (true positive/true positive + false negative) scores (Figure S1B,C, Supporting Information), which constitutes an excellent classification algorithm. These scores are slightly degraded by the fact that not all iPSC cysts exhibit a lumen: when they reach confluence in their alginate shell, an inward cell growth is observed, which fills the lumen.^[^ ^40^ ^]^

Additionally, we correlate these morphological and phenotypical approaches to achieve a more comprehensive description of the MCAs. Using the Beer–Lambert law for light attenuation in an absorbing medium, the thickness *h* of a sample is calculated from the measurement of the transmitted light intensity *I* using *h* = *h*_0_ × log(*I*/*I*_0_), where *h*_0_ is the attenuation length of the medium, and *I*_0_ is the incident optical intensity. Determining *h*_0_ has been shown to bear information on the chemical composition of a tissue, which makes it useful in diagnostics.^[^ ^41^, ^42^ ^]^ Here, we estimate *h*_0_ by fitting the value of log(*I*/*I*_0_) to the diameter *D* of HEK spheroids with a linear model. Although the ellipsoid nature of spheroids introduces a geometrical bias, we observe a good fit at the population level, as shown in Figure 2F. We measure *h*_0_ = 1.4 ± 0.1 × 10^2^ µm, which is in agreement with the typical value of 1.3 × 10^2^ µm measured for kidney tissue.^[^ ^43^ ^]^ Though the optical properties of a tissue depend on the cell types it comprises, we also use this model to estimate the thickness of the cell monolayer of iPSC cysts with the value of *h*_0_ previously determined (Figure 2G). At the center, assuming the attenuation in the lumen is negligible, the estimated thickness *h* should be equal to twice the thickness of a cell. We derive the scaling law *h* = *D*/1.62, which indicates that cysts with a larger diameter have a thicker cell monolayer (Figure S1D, Supporting Information). Remarkably, previous studies have shown that iPSC cysts exhibit an unusual anisotropic growth where the monolayer thickens as the cysts grows, which supports the validity of our results.^[^ ^44^ ^]^ Overall, using this combined pheno‐morphological analysis in large populations of MCAs yields quantitative information on complex physiological mechanisms which could hardly be detected with the analysis of only a few samples.

### 2.3 Continuous Flow of MCAs and Encapsulation in Millimetric Drops

**Figure 3:**
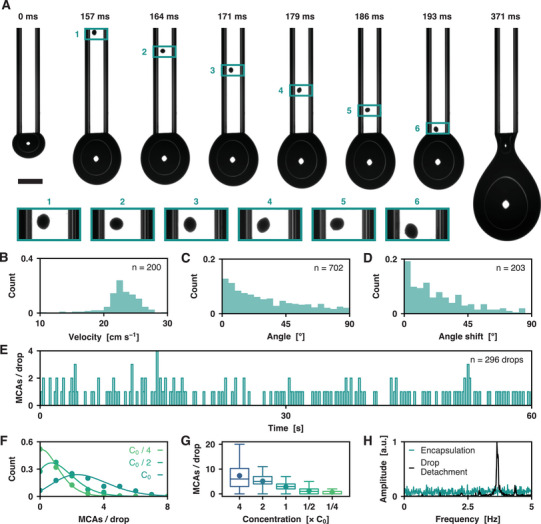
Controlled flow and encapsulation of individual MCAs into liquid drops. A) Principle of the encapsulation step. The micrograph used for image processing is shot right upstream the capillary tip, <10 ms before the encapsulation of the MCA which is instantaneously found in the drop after its detection. Scale bar: 1 mm. B) Histogram of the velocities of MCAs inside the capillary. *n* = 200 spheroids. C) Histogram of the angles formed by the direction of the flow (*z*‐axis) and the major axes of the MCAs, obtained with an ellipse fit. *n* = 702 spheroids. D) Histogram of the angle shifts of the MCAs along their trajectory in the capillary. Only 17% of the spheroids rotate by more than 45°. *n* = 203 spheroids. E) Chronogram of the number *N*_s_ of MCAs per drop. The data shows drop‐to‐drop variations as well as variations over longer time scales. *n* = 296 drops. F) Distributions of *N*_s_ for different concentrations of MCAs. Data points are represented as colored dots. Solid curves with the corresponding colors represent fits of a Poisson statistic, where the *λ* parameter is taken equal to the mean *N*_s_ value for each distribution. *n* = 200 drops for each distribution. G) Box plot of the distributions of *N*_s_ as a function of the concentration, normalized to a reference concentration *C*_0_ = 20 MCAs per mL. Solid points represent the *λ* values. *n* = 200 drops for each distribution. H) Time‐domain discrete Fourier transforms of the times of drop detachment and MCA encapsulation signals. The drop generation frequency distribution is peaked at 3.8 Hz while only randomness is observed in the MCA encapsulation signal.

The encapsulation of MCAs into liquid drops is achieved by flowing them into a square glass capillary (**Figure** **3**A). The choice of a capillary with a cross‐section larger than twice the diameter of the MCAs (800 × 800 µm^2^) mitigates the risk of clogging and flow instabilities. In contrast to most chips used to manipulate individual cells, the capillary is placed vertically to prevent sedimentation of the aggregates, which reduces the likelihood for them to stick to the inner walls of the capillary.

MCAs in suspension in a culture medium solution are gently flowed through a polytetrafluoroethylene (PTFE) tubing and through the capillary using a pressure pump at a constant flow rate of typically *Q* = 100–200 µL s^−1^ in a laminar flow regime (*Re* = 10^2^). As shown in Figure 3B, the velocity of the MCAs in the capillary is of the order of 10 cm s^−1^. This yields a shear stress *τ* = 10^−1^ Pa, to which MCAs are exposed during typically 1 s. This is far below the 10^3^ Pa threshold required to trigger mechanotransduction signals which may eventually affect cell viability.^[^ ^45^ ^]^ In this laminar flow, MCAs tend to orient themselves parallel to the longitudinal axis of the capillary and they hardly rotate along their motion in the flow, as shown in Figure 3A,C,D. The major and minor axes of the MCAs are overall aligned with the imaging plane, which leads to negligible errors in the calculations of the MCAs’ dimensions.

In this range of flow rate, the system is in a dripping regime where millimetric drops of culture medium are produced with a constant volume *V*_d_ = 50 µL, or equivalently a constant radius *R*_d_ = 2.3 mm. Each MCA arriving at the end of the capillary falls into the current pendant drop before its detachment. Although a fraction of each drop remains attached to the capillary (typically 5% volume fraction), we could not observe a single instance of an MCA in this remaining fraction. This ensures that when an MCA is analyzed right prior its exit of the capillary, it is found in the very next drop and not in the one that follows it, which would complicate the sorting step.

A drop‐based sorting system requires control over the number of objects encapsulated in each drop to minimize the probability of co‐encapsulation. Here, it requires to prevent the sedimentation of MCAs in the injection vial by gently agitating it. We characterize this encapsulation step by measuring the distribution of *N*_s_, the number of MCAs found in each drop (Figure 3E and Movie S1, Supporting Information). At a reference concentration of *C*_0_ = 1000 MCAs in 50 mL of culture medium, *N*_s_ randomly varies between 0 and 9 MCAs per drop, independent of the contents of the preceding drop. In a homogeneous suspension, *N*_s_ is expected to follow a Poisson statistic. The probability *P* of finding *N*_s_ aggregates in a drop is therefore given by $P\left(N_{S}\right)=e^{-\lambda }\;\lambda^{N_{s}}\;/\;N_{s}\;,$ where *λ* = *C* × *V*_d_ is the average number of objects per drop.^[^ ^46^ ^]^ In Figure 3F,G, we vary the initial concentration *C* of MCAs and plot the corresponding distributions of *N*_s_. We observe an excellent agreement of the experimental data with theoretical Poisson distributions. Though this statistic does not provide deterministic control over the number of MCAs in each drop, we use it to estimate the number of co‐encapsulation events. For instance, setting *λ* = 0.1 results in 90% empty drops and <0.5% co‐encapsulations, which leaves ≈10% drops containing a single object.^[^ ^37^ ^]^ However, with *V*_d_ = 50 µL, this configuration imposes a concentration *C* = 2 MCAs per mL, which would reduce the sorting throughput and waste large amounts of culture medium. We instead opt for a typical concentration of 5 MCAs per mL to reduce the processed volume and increase the throughput up to 1 MCA per s. The residual co‐encapsulations cases (≈10%) are mitigated by not sorting the corresponding drops, as detailed in Section 2.5.

Additionally, we emphasize that conveying MCAs within the capillary has no effect on the regularity of the drop production rate. Figure 3H shows the relative amplitudes of the discrete Fourier transforms of the drop pinch‐off timestamps and of the times of arrival of MCAs at the exit of the capillary. While MCAs arrive at random timestamps, drops are generated at a constant frequency of 3.8 Hz, independently of the number of MCAs encapsulated. This regularity legitimates the choice of a fixed delay time between the detection of a target MCA in the capillary and the actuation of a trigger signal to deflect the drops.

### 2.4 Acoustic‐Driven Actuation of Millimetric Liquid Drops

In conventional flow cytometers, drops in the *R*_d_ = 100 µm radius range are actuated using the electrostatic force by applying a constant electric field perpendicular to the free‐fall trajectory of the drops.^[^ ^26^ ^]^ However, since this force scales as *R*_d_^2^, the electric field should be increased by more than two orders of magnitude for millimetric drops, typically reaching over 10^5^ V m^−1^. This would lead to the breakup of the drops, hence forming an aerosol.^[^ ^47^ ^]^ On the contrary, placing a millimetric liquid drop in a standing‐wave acoustic field yields an ARF strong enough to manipulate the drop on a surface,^[^ ^48^ ^]^ and even to keep it in acoustic levitation.^[^ ^49^ ^]^ Physically, a standing‐wave acoustic field is scattered by the presence of a liquid drop, which creates second‐order pressure and velocity fields. These secondary fields have non‐null time averages and thereby contribute to a net force *F*_a_ on the surface of the drop, parallel to the direction of propagation of the incident acoustic waves.^[^ ^50^, ^51^ ^]^ When *R*_d_ is small relatively to the acoustic wavelength *λ*_a_, this acoustic radiation force writes:

(1) $$F_{a}=2\pi^{2}\phi \frac{R_{d}^{3}p_{a}^{2}}{\lambda_{a}\rho_{0}c_{0}^{2}}\;$$

where *p*_a_ is the acoustic pressure amplitude, *ρ*_0_ is the density of air, *c*_0_ is the speed of sound in air, and *Φ* ≈ 5/6 is the acoustic contrast factor between air and water.^[^ ^52^ ^]^ *F*_a_ is directed from the pressure antinodes toward the pressure nodes. This model yields a good approximation, as long as *R*_d_/*λ*_a_ < 0.3.^[^ ^53^ ^]^ The scaling of *F*_a_ with the volume of the drop is what makes it suitable for the manipulation of large drops against gravity.

**Figure 4:**
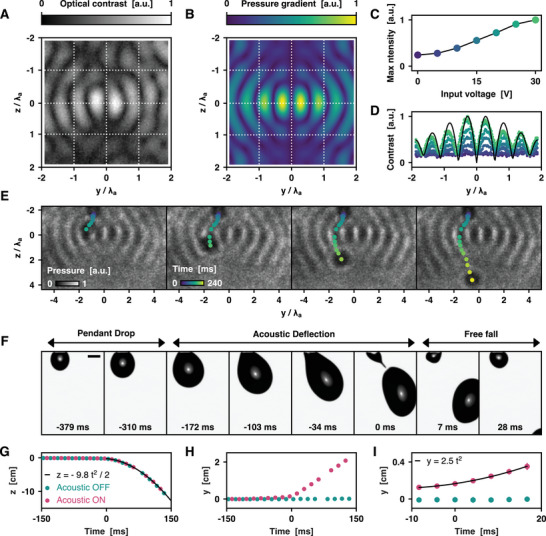
Deflection of millimetric drops using the ARF. A) Schlieren deflectometry data of the standing‐wave acoustic field generated by the two spherical caps of ultrasonic transducers. The optical contrast is proportional to the gradient of the pressure. B) Numerical simulation of the gradient of the pressure along the *y*‐axis. C) Plot of the maximum pixel intensity versus acoustic input voltage. The response of the system is linear in the 10–25 V working range. D) Plot of the optical contrast versus *y* coordinate with *z* = 0. Colored points represent Schlieren data with the input voltage color‐coded accordingly to plot C). The solid black curve corresponds to the numerical simulation in plot B). E) Shadowgraphy images of a drop falling in the acoustic field, where the pixel intensity is proportional to the pressure. The drop follows the acoustic antinodes lines (dark pixels). F) Images of the behavior of a pendant drop in the standing‐wave acoustic field. Scale bar: 1 mm. G) Plot of the trajectory falling drops along the *z*‐axis. The acoustic actuation has no influence on the drops’ trajectories in this direction, which ensures stable drop production. H) Plot of the trajectory falling drops along the *y*‐axis. The acoustic actuation yields a horizontal displacement *Δy* = 2 cm over a falling distance *Δz* = 10 cm. I) Zoom‐in of plot H). We fit Newton's second law of motion with an ARF of constant intensity *F*_a_, yielding *F*_a_ = 2.5 ± 0.2 × 10^−4^ N. In G–I), dots represent the mean positions of the drops’ centroids (acoustic ON: *n* = 11 drops, acoustic OFF: *n* = 6 drops).

We generate a standing‐wave pressure field along the *y*‐axis with two hemispherical arrays of 36 ultrasonic transducers in phase operating at 40 kHz (Figure 1). This sets *λ*_a_ = 8.6 mm with *R*_d_/*λ*_a_ = 0.25, and the resulting standing‐wave acoustic field has an inter‐node distance *λ*_a_/2 = 4.3 mm. We characterize the geometry of the acoustic field thus generated by comparing Schlieren images and numerical simulations (**Figure** **4**A,B). In Schlieren deflectometry, the gradients of refractive index induced by the presence of pressure gradients in the acoustic field leads to the bending of light rays coming with orthogonal incidence—the experimental system is described in Figure S3 (Supporting Information). Using a high‐speed camera recording 400 images at 39 kHz, we obtain a dynamic representation of the oscillations of the acoustic field over 10 acoustic periods (see Movie S2, Supporting Information). We measure the optical contrast, defined as the peak‐to‐peak pixel intensity amplitude over a period, to reconstruct the map of the acoustic field. The resulting image matches with the numerical simulation of the acoustic field, which we computed using a previously described method, detailed in the Experimental Section.^[^ ^49^, ^54^, ^55^ ^]^ Theoretical developments show that the optical contrast obtained in Schlieren deflectometry is proportional to the gradient of the pressure field integrated along the optical axis.^[^ ^56^ ^]^ We verify this in Figure 4C,D, which further confirms the linear response of our acoustic setup.

While the levitation of millimetric drops in similar acoustic devices was extensively described,^[^ ^49^, ^54^ ^]^ the behavior of drops falling in a standing‐wave acoustic field was not explored yet. We first sought to visualize the trajectories of falling drops respectively to the pressure nodes and antinodes using direct shadowgraphy, a variant of Schlieren deflectometry where the optical contrast is directly proportional to the Laplacian of the pressure field, instead of its gradient.^[^ ^56^ ^]^ Here, as shown in Figure 4A–D, the pressure field is appropriately modeled with sinusoidal waves, in which case the Laplacian of the pressure is proportional to the pressure field itself. Therefore, the shadowgraphy method allows direct visualization of the pressure field. Unfortunately, it is based on out‐of‐focus imaging, which do not enable to set a precise *yz* scale, hence the requirement for a combined use of Schlieren and shadowgraphy for complete characterization of the pressure field. Movie S3 (Supporting Information) and Figure 4E show that drops actually follow the pressure node lines along their fall, and therefore have a curvilinear trajectory. In this configuration, it is not possible to impose a net horizontal displacement to the drops, as they are deflected along the *y*‐axis, and then in the opposite direction with the same amplitude. It is therefore necessary to place the tip of the capillary right below the *z* = 0 plane to break the symmetry of the motion. The pressure field is also symmetrical with respect to the *y* = 0 plane, which corresponds to a pressure antinode.^[^ ^55^ ^]^ For this reason, the exit of the capillary needs to be placed slightly off‐center, typically *δy* = *λ*_a_/8, so that the drops fall between a node and an antinode where the ARF is stronger.

Figure 4F shows the deflection of a pendant drop upon activation of the acoustic field during its detachment, which occurs in three phases. First, the pendant drop remains attached to the capillary under the action of surface tension force *F*_γ_. As it grows due to the constant flow of fluid through the capillary, it reaches a critical radius *R*_d_ *^γa^* = (*γλ*_a_/*F*_a_)^0.5^ given by the *F*_γ_/*F*_a_ balance. Above *R*_d_ *^γa^*, the ARF overcomes the capillary forces in the *y*‐direction, which deflects the drop horizontally. After reaching a second critical radius *R*_d_ *^γg^* = (*γ*/*ρg*)^0.5^ given by the *F*_γ_/*F_g_* balance where *ρ* = 10^3^ kg m^−3^ is the density of water and *g* the gravitational acceleration constant, the pendant drop eventually detaches from the capillary with a net momentum along the *y*‐axis.

To compare the dynamics of deflected and non‐deflected drops along their fall, we analyze the trajectories of their centroids (Figure 4G–I and Movie S4, Supporting Information). We first observe that the deflection of the drops is initiated before their detachment with excellent reproducibility. When operating at a rate of 2 drops per s, the ARF imposes a lateral deflection *Δy* = 1 cm in about *Δz* = 3 cm falling distance. This is sufficient to collect deflected drops and non‐deflected drops in separate vials. We successfully separate individual drops at flow rates up to 500 µL s^−1^, or equivalently 10 drops per s, which corresponds to the dripping/jetting transition.

A more detailed analysis of the trajectories provides information on the intensity of the ARF acting on large, deformable liquid drops, which could otherwise only be calculated at the cost of subtle corrections that are beyond the scope of the present work. We first track the coordinates of the drops’ centroids over time to obtain the vertical and horizontal displacements *z*(*t*) and *y*(*t*) (Figure 4G,H). Strictly speaking, *F*_a_ is the component of the ARF along the axial *y*‐axis, but the ARF also has components in the radial *x*‐ and *z*‐directions.^[^ ^52^ ^]^ However, we neglect them in a first order approximation, as they are reportedly ≈5 times weaker than the axial component *F*_a_.^[^ ^53^ ^]^ In our system, the *x* component is cancelled out anyway since the capillary is placed in the *x* = 0 plane of symmetry. We make the bold simplification that *F*_a_ is approximately constant in space and time along the drop's trajectory along the pressure nodes, which is coherent with the acoustic maps shown in Figure 4A,B. Under these assumptions, the initial speed at the exit of this zone of actuation is simply *v_0_* = *F*_a_ *Δt_a_* / *m*, where *Δt_a_* is the time of flight of the drop in the acoustic field and *m* = 5 × 10^−5^ kg is the mass of the drop. In the absence of friction, the drop is in free fall after exiting the acoustic field and *z*(*t*) *= gt^2^*/2. Figure 4G shows an excellent agreement of our experimental data with this simple kinetic model, which legitimates the omission of the *z* component of the ARF. From the fit of *y*(*t*), we obtain *F*_a_ = 2.5 ± 0.2 × 10^−4^ N ≈ *mg*/2. This force is too weak to quantitatively deflect a falling drop that has already reached its terminal velocity, but here a strong initial deflection of ≈40° is observed because it occurs while the drop has little to no vertical speed. Using Equation (1) to estimate the acoustic pressure in the region of deflection, we obtain *p*_a_ = 1.2 × 10^3^ Pa or 1.6 × 10^2^ dB, which is on par with values previously reported for similar acoustic systems.^[^ ^49^, ^53^, ^54^, ^55^ ^]^ This relatively intense acoustic field is however almost completely reflected at the surface of the drop due to the high contrast in acoustic impedance between air and water. The encapsulated MCAs are therefore protected from potential interaction with the acoustic field.

### 2.5 Sorting Large Populations (*n* > 1000) of MCAs in Continuous Flow

**Figure 5:**
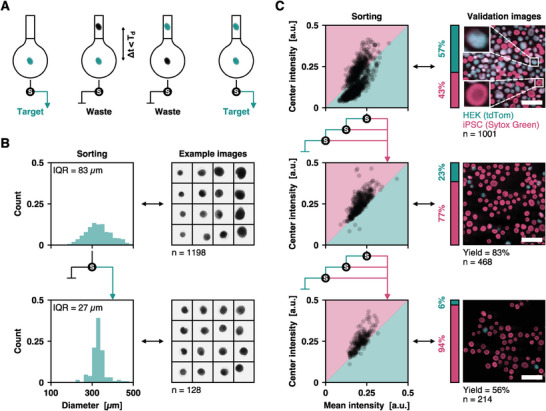
Sorting performance of ImOCAS. A) Illustration of the co‐encapsulation mitigation strategy. Drops containing at least one nontarget object are discarded: if two objects or more are detected within *Δt* < *T*_d_ and one of them is classified as waste, the following drop is not sorted. B) Histograms of distributions of sizes and typical images of HEK spheroids before and after sorting them using two threshold radii *D*_min_ = 320 µm and *D*_max_ = 336 µm. The radius distribution is initially broad before sorting (top row, *IQR* = 83 µm, *n* = 1198 spheroids) and is narrowed down after sorting (bottom row, *IQR* = 27 µm, *n* = 128 spheroids). The median diameter *M*_D_ = 329 µm remains unchanged. Not all spheroids fit in the [*D*_min_ *, D*_max_] range because the definition of *D* depends on the orientation of a spheroid with respect to the camera, hence highly nonspherical spheroids are imperfectly characterized by the measure of *D*. Images are 800 × 800 µm^2^. C) Sorting strategy and validation fluorescence images for the sorting of iPSC cysts from a sample where they are mixed with HEK spheroids. We use the Center intensity > Mean intensity criterion to detect target cysts, as shown by the shaded regions in the left column plots. The fluorescence images shown in the right column are acquired afterwards to measure the purity *P* and yield *Y* sorting metrics: *P* = 77% and *Y* = 83% after one sorting step, *P* = 94% and *Y* = 56% after two sorting steps. Scale bars: 1 mm. The *S* disks represent one sorting step and the arrows indicate the sorting procedure. Top row: *n* = 1001 MCAs; middle row: *n* = 468 MCAs, bottom row: *n* = 214 MCAs.

After optimization of the detection, encapsulation, and actuation modules, efficient sorting simply requires the synchronization of these steps. Here, the suspension of MCAs is flowed at constant rate, setting a drop generation frequency of 3.8 drops per s with a period *T*_d_ = 0.26 s between successive drops. Since the images are acquired at ≈1 mm above the capillary exit where the MCAs flow at ≈10 cm s^−1^ an MCA is found in the drop ≈0.01 s after being detected. Therefore, upon detection of a target MCA, only the very next drop must be sorted, which is achieved by a command signal sent to the waveform generator to activate the acoustic field only for a duration of 1.5 × *T*_d_, right after the image processing returns a positive result. Additionally, we implement a simple decision tree to minimize potential co‐encapsulation events that could deteriorate the sorting efficiency (**Figure** **5**A). When two objects are detected within a duration *Δt < T*_d_, the next drop is not deflected if one or more of the objects is identified as nontarget.

One key challenge in large‐scale biological assays is to ensure homogeneity within the sample population. For instance, to evaluate the impact of a drug in a population of spheroids, their initial size distribution must be uniform to avoid the bias of potential size‐dependent activity of drugs. Thus, we first evaluate the efficiency of ImOCAS by sorting spheroids based on their size. We compare the diameter *D* of a spheroid measured in an image with two threshold radii *D*_min_ and *D*_max_ set by the user. Due to the nonspherical shape of a spheroid, the definition of its radius slightly depends on its orientation with respect to the imaging plane, which introduces variability in the determined value. The objective of this sorting step is therefore to reduce the width of the size distribution to obtain a homogeneous sample, rather than to accurately select a size range. As shown in Figure 5B, we first use ImOCAS in a cytometry mode to characterize our starting population of *n* = 1198 spheroids with a median value *D*_M_ = 329 µm, and an interquartile range *IQR* = 83 µm. The whole distribution of *D* extends between 117 and 558 µm. We define two thresholds *D*_min_ = 320 µm and *D*_max_ = 336 µm that encompass 10% of the spheroids centered around the median diameter. We then use ImOCAS to sort the spheroid population accordingly, and we finally use it to screen the resulting sample. After sorting, the width of the size distribution is considerably reduced, with *IQR* = 27 µm, without modifying the median diameter *D*_M_. This demonstrates the efficiency of ImOCAS to sort spheroids based the measurement of a continuous morphological parameter (here, their diameters).

ImOCAS is also able to sort MCAs based on their phenotypical signature, which finds applications in organoid screening. For instance, in the case of lumenized iPSC cysts, live samples clearly exhibit a lumen while differentiated tissues resemble plain spheroids.^[^ ^40^ ^]^ More broadly, the coexistence of cyst‐like and spheroid‐like phenotypes has previously been reported in vitro,^[^ ^57^ ^]^ and the transition from cyst to spheroid was shown to be a marker of malignant traits in mammary glands models.^[^ ^58^ ^]^ Here, we mimic this situation using a heterogeneous sample containing *n*_c_ = 432 of iPSC cells (target) stained with Sytox Green and *n*_s_ = 569 spheroids of HEK cells (waste) constitutively expressing tdTom at their membranes. The sorting is again performed based on bright‐field image analysis, but we use fluorescent dyes to subsequently validate the sorting results via epifluorescence imaging. We use the Center intensity > Mean intensity criterion as a signature of the presence of a lumen to sort the two sub‐populations (Figure 5C). Since drops containing two or more MCAs are not sorted, we compensate for the expected decrease in yield with a multiple‐step sorting approach. After sorting the initial sample population, the contents of the waste vial are re‐sorted to recover the discarded target objects, and finally the contents of the resulting waste vial are also sorted. To be selected, an MCA therefore needs to be marked once as a target among three sorting steps (Figure 5C, rows 1 and 2). We assess the sorting quality by measuring the purity *P = n*_c_ */n*_s_ and the yield *Y = n*_c_^S^ */n*_c_, where *n*_c_^S^ is the number of sorted cysts. Starting from *P* = 43%, this first sorting step removes 75% of the spheroids while keeping *Y* = 83% of the cysts to obtain *P* = 77%. The discrepancy between this satisfactory purity rate and the excellent precision of the image processing algorithm mainly emerges from the aggregation of some MCAs into clusters of two or more objects, which cannot be properly classified. However, since the whole sorting procedure is short (<1 h), it is easy to further increase the purity with a second sorting step, leading to *P* = 94% (Figure 5C, bottom row).

Overall, ImOCAS is suitable for sorting large populations of MCAs based on both morphological and phenotypical parameters at a high throughput, typically 100 times faster than manual sorting.

## 3 Discussion

The use of 3D cell models in both fundamental biology and biomedical applications has long been hampered by the absence of robust protocols to produce and analyze these complex objects. In the last 20 years, numerous methods based on microfluidics and microfabrication techniques have emerged and now allow the reproducible, high‐throughput production of MCAs. However, to perform statistically relevant biological assays on a homogeneous population of MCAs, one also needs to sort them to reduce the inherent morphologic and phenotypic variability of these biological samples. There is therefore a growing need to implement strategies for analyzing and sorting large populations of MCAs. Counter‐intuitively, simply scaling up the microfluidic devices that were designed to manipulate single cells is highly challenging, mainly due to the prominence of inertia and gravity at the scale of MCAs (50 µm–5 mm).

To the best of our knowledge, flow‐based organoid and spheroid sorting using image analysis has only been achieved by a handful of commercial systems and, recently, one research team.^[^ ^38^, ^59^, ^60^ ^]^ However, these systems are limited by the impossibility to tailor them for custom applications, by their dependence only on pre‐defined morphological parameters or on averaged intensity levels rather than comprehensive image analysis, and/or by their incompatibility with large populations exceeding thousands of MCAs.

To address these limitations while optimizing sorting speed and analysis refinement, we developed ImOCAS, a drop millifluidic system which performs automated screening and sorting experiments on large populations of MCAs. Like a traditional flow cytometer, ImOCAS operates in three successive steps: detection of a feature in MCAs in flow, encapsulation of single MCAs in liquid drops, and actuation of the drops of interest. Here, each step is sought to be adapted to the manipulation of MCAs instead of single cells. First, the morphological and phenotypical signatures of MCAs are detected using rapid image processing while the MCAs flow inside a glass capillary. Then, individual MCAs are encapsulated in millimetric drops of culture medium in a dripping mode. Finally, the drops are actuated using the ARF upon the generation of a standing‐wave acoustic field in the vicinity of the drops.

The main strengths of ImOCAS are its versatility regarding the detection parameters and its potential to be used in any biology laboratory since it does not require expensive nor complex apparatus other than a computer with standard performance for image analysis and a fluid control system. Its small size (40 × 20 × 20 cm^3^) makes it fit inside a biosafety hood when working in a sterile environment is required. The choice of a label‐free imaging method also makes it compatible with live samples and greatly accelerates the sample preparation procedures. Biological samples can typically remain outside an incubator for at least 2 hours, which enables the processing of more than 6000 MCAs in one run with ImOCAS, including the sample dilution step—equivalent to six million cells that can subsequently be used. Within this context, the current sorting throughput of one MCA per second cannot be directly compared to the kilohertz rates reported for single‐cell sorting, given the 1000‐fold volume ratio between the sorted objects. Indeed, a usual end‐goal is to perform biochemical assays for quantitative screening (e.g., multi‐omics analysis), requiring batches of around 1000 cells, which is where the demand for ultrahigh‐throughput single‐cell sorting stems from. In this work, we achieve comparable screening potential since each MCA typically contains hundreds of cells.

In ImOCAS, the sorting throughput is only limited by the frequency of drop production, which could be increased by reducing the size of the drops. This may be achieved using a piezo‐electric actuator to vibrate the capillary and thereby trigger the detachment of the drops, for instance. Further optimizing the flow upstream of the capillary to increase the fraction of non‐empty drops constitutes a promising approach, which was already observed with the encapsulation of smaller particles in microfluidic chips.^[^ ^61^ ^]^ On the detection side, although ImOCAS already allows a thorough pheno‐morphological analysis of MCAs, modifications could be made to meet specific requirements. For instance, the use of a 3D imaging method would offer a more comprehensive representation of the structural complexity of MCAs. However, this poses technical challenges as ImOCAS requires usage of a long working distance objective in order not to interfere with the acoustic field in the deflection zone. In the actuation step, there is no physical limitations to the use of more complex acoustic fields to sort drops in more than two categories. In particular, the acoustic nodes may be accurately re‐positioned in real‐time with a simple phase shift between the two arrays of transducer, hence changing the direction of deviation along the *y*‐axis. Overall, ImOCAS is here presented in its most general form and could be easily tailored for custom applications.

ImOCAS may rapidly be adopted in biology facilities as a robust approach to analyze and sort complex 3D biological models. Thanks to its versatility and its simplicity, it may be applied broadly to standardize drug testing experiments in MCAs by first ensuring the homogeneity of the tested MCAs and then screen the results. It may also be used to detect rare events that would be left unnoticed when analyzing only a small number of MCAs. We anticipate that the compatibility of ImOCAS with virtually any organoid and spheroid samples will unveil new insights in 3D biology, in both fundamental and applied research.

## 4 Experimental Section

### 2D Cell Culture

HEK cells obtained from the Bordeaux Institute of Oncology were infected with a lentivector backbone expressing tdTom with Nter Palmitoylation (under the control of hPGK), using standard methods. Infected cells constitutively express the tdTom fluorescent molecule at their membrane. Cells were then cultured in culture‐treated plastic flasks (Corning, cat. no. 353 136) and maintained in Dulbecco's modified Eagle medium (DMEM) (Biowest, cat. no. L0103‐500) supplemented with 10% fetal bovine serum (Capricorn, cat. no. FBS‐16A), 5% penicillin‐streptomycin (Gibco, cat. no. 15 140 122). iPSC cells purchased from Corriell Institute (AICS‐0023) were cultured in culture‐treated plastic flasks (Corning, cat. no. 353 107) coated with 5% Matrigel (Corning, cat. no. 354 234), maintained in mTeSR Plus (StemCell, cat. no. 100–0276). Both cell types were maintained under water‐saturated 5% CO_2_ atmosphere at 37 °C.

### Formation of MCAs

Cell aggregates were formed using the CCT method, previously described in details.^[^ ^13^ ^]^ Briefly, cells were detached from their flasks using trypsin (Biowest, cat. no. L0930‐100) for HEKs and Accutase (StemCell, cat. no. 0 7920) for iPSCs. Cells were resuspended in their appropriate culture medium and mixed with Matrigel (Corning, cat. no. 356 234) at 50% volume fraction. The resulting solution was injected in a 3D printed microfluidic chip together with a solution of sorbitol 300 × 10^−3^ m (Sigma–Aldrich, cat. no. S1876) and a solution of 2% alginate (AGI, cat. no. I3G80) to form a composite liquid jet consisting in a triple co‐flow of the cells/Matrigel mix, sorbitol and alginate solutions. The jet fragmentation resulted in the formation of alginate shells, collected in a calcium bath to trigger alginate reticulation, hence forming solid core–shell structures with cells embedded in Matrigel in the core. The seeding density ranged from 10 to 100 HEK cells per shell, and 1–10 iPSC cells per shell. In the case of iPSCs, ROCK inhibitor (Tocris, cat. no. 1254/10, 10 × 10^−3^ m) was added and replenished at 1/1000 dilution every day for the first 48 h after formation of the shells.

Since alginate is optically transparent, it does not alter the image‐based analysis of the aggregates, and this work effectively probes only the morphology and size and the MCAs contained inside the alginate shells. Any other classical method to produce MCAs may be used, provided that the aggregates can be resuspended in a solution.

### 3D Cell Culture

Cell‐laden alginate shells were cultured in the appropriate culture medium for 7 days, with medium replacement every 2–3 days. The cells were then fixed in a solution of 4% paraformaldehyde (Sigma–Aldrich, cat. no. 47 608) overnight and stored in glucose free and phenol red free DMEM (Gibco, cat. no. A1443001) at 4 °C. The nuclei of iPSC cells were stained with SYTOX Green after fixation (NucGreen Dead 488, Thermo Fisher).

### Image Acquisition

In the glass capillary, cell aggregates were illuminated with a white light LED (MWWHL4, Thorlabs), collimated with a single convergent lens (AC254‐050‐A‐ML, Thorlabs). The transmitted light was collected through a zoom lens (1‐60135 6.5×, Navitar) and acquired with a CMOS monochromatic camera (acA720‐520um, Basler) at ≈100 fps. The typical image size was 256 × 256 px^2^ for a field of view of 800 × 800 µm^2^, yielding ≈3 µm px^−1^ (i.e., 6 µm resolution).

### Image Processing

On‐the‐fly image processing was conducted using a Python script with the Numpy, OpenCV, Skimage, and Scipy libraries. First, the intensity of each pixel was normalized against that of an image without objects. Subsequently, an intensity threshold was applied to create a mask containing the low‐intensity pixels. Images where the number of pixels in the mask was below a set value were immediately discarded, while the other images were further analyzed. For these images, the centroid of the mask was calculated, and a 11‐px radius disk was drawn around it to define the Center region. By eroding the mask by 6 px, the Inner region was obtained, and the difference between the initial region and the Inner region yielded the Border region. Mean intensities across each region were calculated by summing pixel intensities and dividing by the area of the region. Elliptical fits were performed using the regionprops function from Skimage.

### Millifluidic Injection

Cell aggregates were flowed from a standard Falcon tube (catalog no. 352 098, Corning) into an 800 × 800 µm^2^ square glass capillary (VitroCom, cat. no. 8280) through a PTFE tubing with 1.06 mm internal diameter (Fisher Scientific, cat. no. 11 949 445). The connection between the tubing and the capillary was made using PDMS (Sylgard 184, Dow Corning). A mold was fabricated by sticking a capillary onto a glass slide, then pouring the PDMS on top of it before baking it at 60 °C for 4 h.

### Fluid Control System

The aggregate suspension was transferred into the encapsulation chip using a pressure controller (MFCS‐EZ, Fluigent). The driving pressure was set to ≈40 mbar to achieve flow rates of 100–500 µL s^−1^, or 2–4 drops per s, using a PTFE tubing of ≈30 cm in length. This setup maintained a steady flow rate without requirement for a flow meter.

### Sample Stirring

Due to their size, organoids and spheroids in suspension tend to sediment at the bottom of a 50 mL vial within ≈10 s. It is therefore necessary to mix the suspension continuously. However, using a magnetic stirrer would harm the aggregates. Instead, the vials containing the suspension were mounted onto a servo motor (MG996R, Tiankon‐gRC), controlled by a microcontroller (Uno Rev2, Arduino), to perform back‐and‐forth rotations with an amplitude of approximately 100°.

### Acoustic Field Design

The acoustic deflection system draws inspiration from prior research on acoustic levitation setups.^[^ ^49^ ^]^ The system comprises two spherical caps designed to hold ultrasonic transducers. These caps were 3D printed using a Digital Light Processing 3D printer (D4K Pro, Envisiontec) and photocurable resin (HTM 140 V2, Envisiontec). The radius of curvature of the caps was set to 86 mm, or equivalently to 10 acoustic wavelengths, ensuring an effective focusing effect while maintaining sufficient space to manipulate the capillary between the two caps. Each cap accommodates 36 ultrasonic transducers (MA40S4S, Murata), arranged along concentric rings and electrically connected in parallel. Upon detection of a target MCA, a command was sent through serial communication to a waveform generator (T3AFG30, Teledyne Lecroy) to trigger a 40 kHz square signal. The signal was amplified with an L298N motor driver electronic chip powered at 9.5 V before being transmitted through the transducer circuit. The two spherical caps were aligned to ensure that each transducer on one cap is facing its counterpart on the other, hence generating a standing‐wave acoustic field with spherical iso‐phase planes.

### Schlieren Deflectometry and Direct Shadowgraphy

The Schlieren images of the acoustic field were obtained with a standard double‐pass setup, as described in Figure S3 (Supporting Information). Monochromatic light generated with an LED (M625L2, Thorlabs) was sent through a pinhole (400 µm aperture) toward a spherical mirror of radius *R* = 180 cm, or equivalently a focal length *f* = 90 cm. The acoustic transducers were placed between the pinhole and mirror, and the pressure field bends the incident rays according to Snell's law. The rays reflected by the mirror were send toward a hi‐speed camera (V210, Phantom) via a beam splitter. Between the beam splitter and the camera, a sharp edge (here, a razor blade) blocked half of the incident light, which created an optical contrast on the sensor. The focus was made on the mirror using a tele‐objective lens (EX DG 70–200 mm, Sigma). In direct shadowgraphy, the sharp edge was removed and the image was taken out of focus.

### Numerical Simulations of the Acoustic Field

A Python script was developed to compute the relative pressure amplitudes generated by the arrays acoustic transducers. An *xyzt* grid was created which ranged over *Δx* = *Δy* = *Δz* = 4 × *λ*_a_, and *Δt* = *T*_a_ = 25 µs, with steps *dx* = *λ*_a_/25, *dy* = *dz* = *λ*_a_/50, and *dt* = *T_a_* / 40. For each point of the grid, the contribution of each transducer to the total amplitude was computed using a piston source model, as previously reported.^[^ ^49^, ^54^, ^55^ ^]^ Briefly, each transducer's contribution was modeled with a spherical wave adjusted by a correction factor for propagation directivity. To compare the numerical simulations with deflectometry images of the field's projection along the *x*‐axis, the simulated *x*‐planes were summed. Following this, the peak‐to‐peak amplitudes were computed over one period and the corresponding gradient along the *y*‐axis was determined.

### Positioning the Acoustic field Relatively to the Capillary

The spherical focus of the acoustic field resulted in a maximum pressure amplitude at the system's center. The intensity of the resulting ARF had local maxima located halfway between the nodes and the antinodes of the standing pressure field, which had an inter‐node distance *λ*_a_/2 = 4.3 mm. Therefore, the tip of the glass capillary had to be positioned along the axis of symmetry of the acoustic field, but slightly off‐center (typically *λ*_a_/8) to maximize the local pressure gradient. In practice, the optic and fluidic apparatus were kept still, and the spherical caps were moved along the *y*‐axis until satisfactory drop deflection was observed, and along the *x*‐axis until no deflection was observed in this direction.

### Statistical Analysis

Results are presented as mean ± standard deviation, unless otherwise specified. Sample sizes (*n*) are included in figure captions where relevant. No outliers were excluded from the statistical analysis. Python 3.10 was used for all image pre‐processing and analysis, as described in the Results Section.

## Conflict of Interest

L.R., J.C.B., P.N., and A.B. are the inventors of a patent application covering the principle of ImOCAS. The authors declare that they have no other competing interests.

## Supporting information



Supporting Information

Supplemental Movie 1

Supplemental Movie 2

Supplemental Movie 3

Supplemental Movie 4

## Data Availability

The data that support the findings of this study are available from the corresponding author upon reasonable request.
