# Supplementary material for: Pheno‐Morphological Screening and Acoustic Sorting of 3D Multicellular Aggregates Using Drop Millifluidics
Source: Adv Sci (Weinh). 2025 Jan 10;12(9):2410677. doi: 10.1002/advs.202410677 (PMC11884609; doi:10.1002/advs.202410677)
Supplement: Supplementary file 1 — Supporting Information [file ADVS-12-2410677-s004.docx]

Supporting Information

**Pheno-morphological screening and acoustic sorting of 3D multicellular aggregates using drop millifluidics**

Leon Rembotte*, Thomas Beneyton, Lionel Buisson, Amaury Badon, Adeline Boyreau, Camille Douillet, Loic Hermant, Anirban Jana, Pierre Nassoy*, Jean-Christophe Baret*

Movie S1

Encapsulation of multicellular aggregates (MCAs) into drops of culture medium at the exit of a glass capillary. The concentration is *C* ~ 5 MCAs per mL, which results in *N_s_* = 0.7 MCAs per drop on average, and 10% of drops containing 2 MCAs or more. Speed × 0.25 (100 fps).

**Movie S2**

Hi-speed Schlieren deflectometry movie of the standing-wave ultrasonic field. The movie corresponds to 10 acoustic periods. Speed × 0.001 (39000 fps).

**Movie S3**

Direct shadowgraphy movie of a drop falling in the standing-wave ultrasonic field. Speed × 0.1 (100 fps). No scale bar can be provided for this movie, since the image formed on the sensor of the camera contains the projection of a continuum of planes along the imaging axis.

Movie S4

Deflection of water drops using the Acoustic Radiation Force (ARF) for sorting purposes. The injection glass capillary is placed at the vicinity of the center of symmetry of two hemispherical arrays of ultrasonic transducers operating at 40 kHz. While the transducers are switched off, drops free fall vertically into a collection vial. Upon activation of the transducers, a standing acoustic field is generated, which results in the application of an ARF at the surface of the drop while they still attached to the capillary. After they detach from it, they enter a free falling regime, but with an initial horizontal speed which allows to collect them in a separate vial. Speed × 1 (120 fps).

**Figure S1**


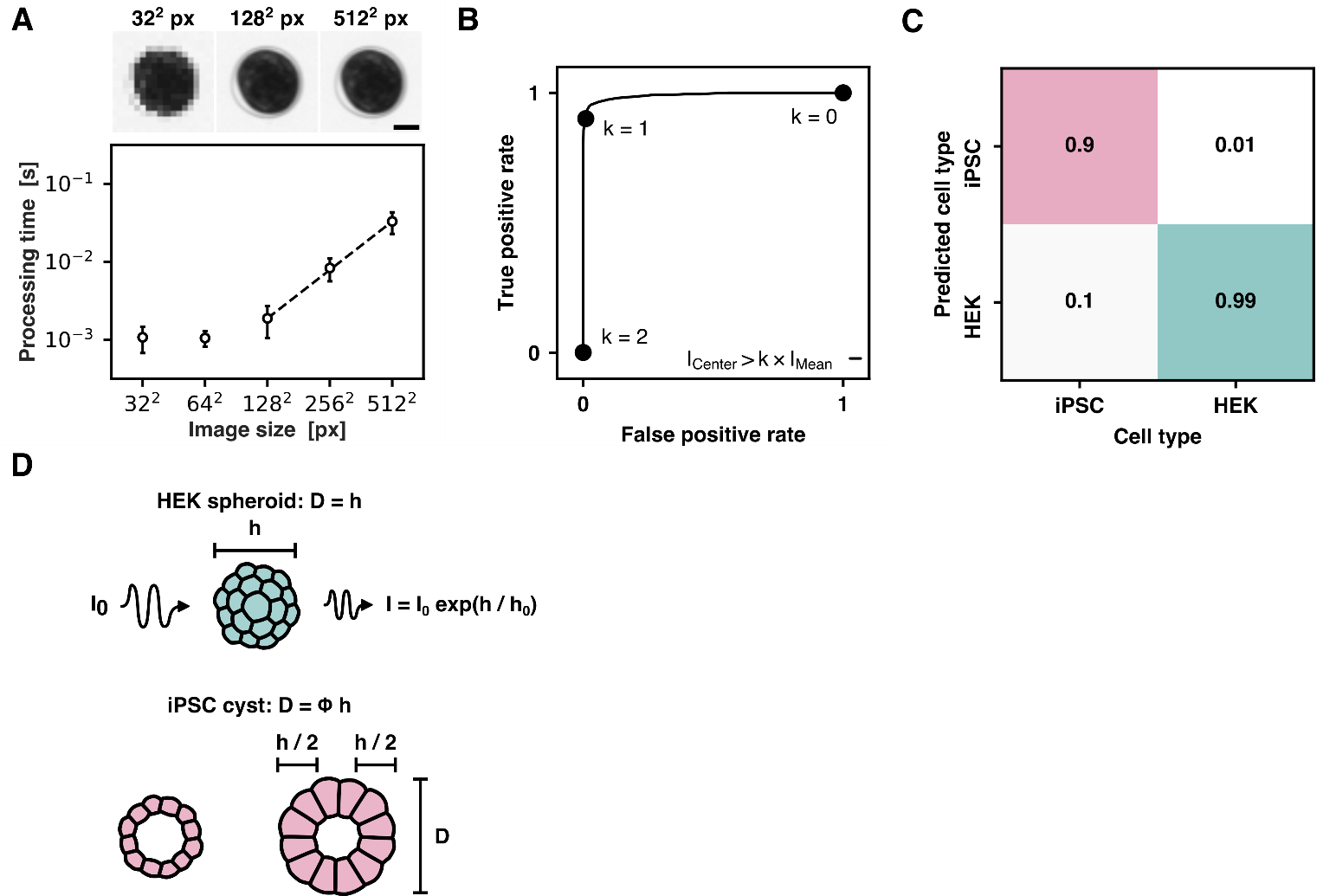


**Figure S1.** Performance of the image processing algorithm. A) Plot of the image processing time vs. image size. Reducing the image size down to 256 × 256 px^2^ results in ~ 3 µm px^-1^ and increases the processing time while keeping satisfactory image definition. Scale bar: 100 µm. Computation times are represented as mean ± standard deviation, computer over a batch of n = 1000 images. B) Receiver operating characteristic curve of the *Center intensity* > *k* × *Mean intensity* classification algorithm. The value *k* = 1 value determined with physical arguments is close to the optimal value (*k* = 0.93). C) Confusion matrix of the classification algorithm using the Center intensity > Mean intensity criterion. The data yields 99% precision and 90% recall scores with true positive and true negative rates of 0.90 and 0.01, respectively. D) Simple model for the estimation of tissue thickness using the Beer-Lambert law of absorption. For spheroids, the thickness is equal to the diameter, which allows to measure the attenuation length *h_0_*. This value is used to estimate the scaling factor *Φ* = *D / h* = 1.62, and the reason why the golden ratio appears here remains a mystery to us.

**Figure S2**


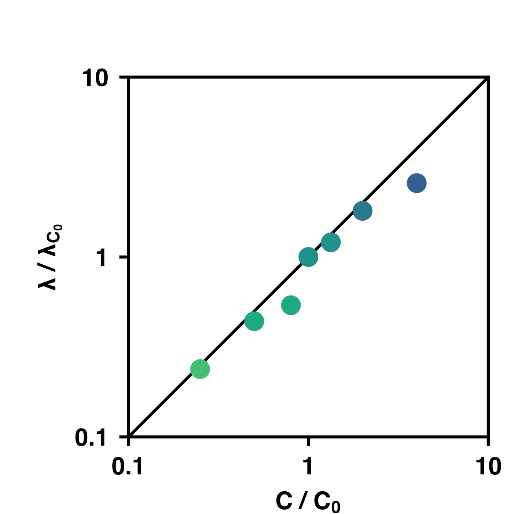


**Figure S2.** Plot of the mean number *λ* of MCAs per drop vs. the dilution factor of the MCA solution. Data is normalized to a reference concentration *C_0_* ~ 20 MCAs per mL. n = 200 drops for each point.

**Figure S3**


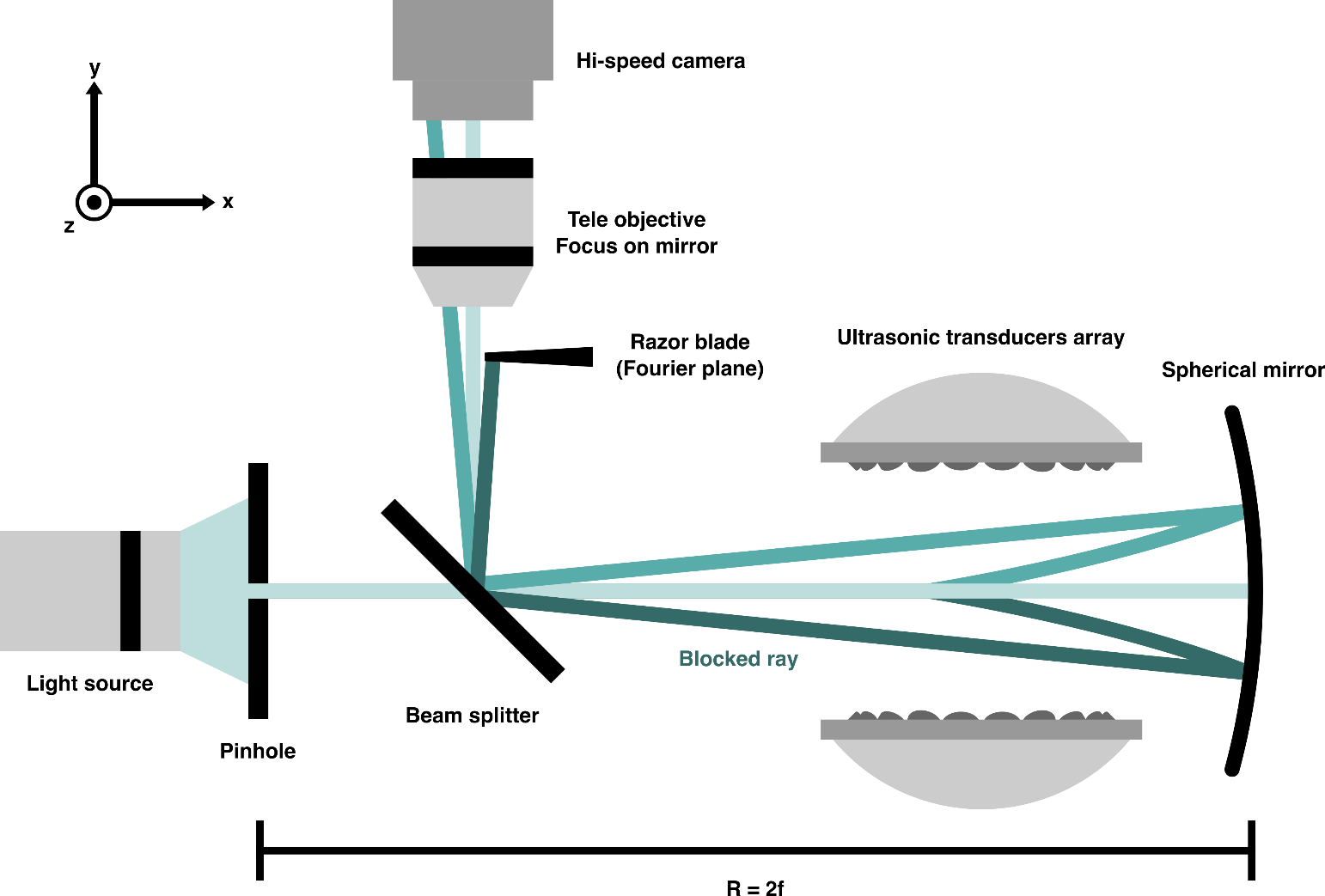


**Figure S3.** Opto-acoustical system for Schlieren imaging. When the acoustic field is activated, the pressure gradients induce a refractive index gradient which deflects the incident light rays. The razor blade located in the Fourier plane of the spherical mirror filter out half of the rays, which creates and optical contrast on the sensor of the camera. For shadowgraphy imaging, the razor blade is removed, the camera is placed out of focus and the projection of the mirrored image forms on the sensor.
